# Powassan Virus Encephalitis after Tick Bite, Manitoba, Canada

**DOI:** 10.3201/eid3009.231344

**Published:** 2024-09

**Authors:** Nathan Smith, Yoav Keynan, Terry Wuerz, Aditya Sharma

**Affiliations:** University of Manitoba, Winnipeg, Manitoba, Canada

**Keywords:** ticks, Ixodes, meningitis/encephalitis, virus, Powassan virus, tickborne, vector-borne infections, Canada

## Abstract

A case of Powassan encephalitis occurred in Manitoba, Canada, after the bite of a black-legged tick. Awareness of this emerging tickborne illness is needed because the number of vector tick species is growing. No specific treatment options exist, and cases with illness and death are high. Prevention is crucial.

On October 2, 2022, a 60-year-old male hobbyist outdoor photographer in southern Manitoba, Canada, noticed a black-legged tick (*Ixodes scapularis*) attached to his neck (Figure). The patient sought treatment for possible Lyme disease and was prescribed doxycycline.

**Figure:**
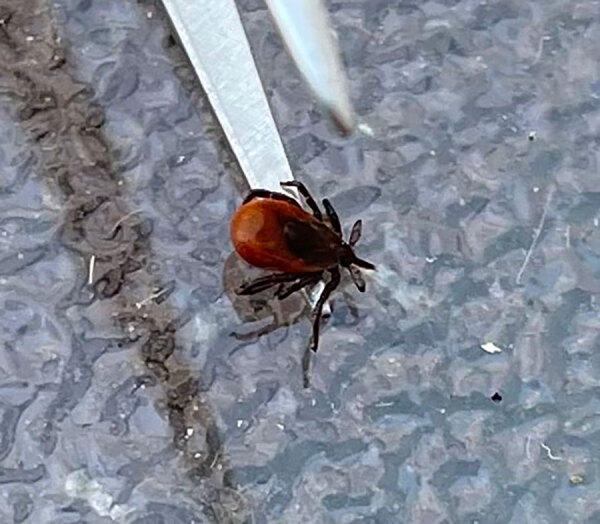
Black-legged tick (*Ixodes scapularis*) after removal with tweezers from a patient in Manitoba, Canada, who was later diagnosed with Powassan virus.

On October 16, 2 weeks after the tick bite, the patient had complaints of diarrhea, nausea, and malaise. He also had a fever that reached 40°C (104°F), a 10–15-pound weight loss, difficulty concentrating, and a bilateral headache, and he became bedbound from weakness and ataxia. He was admitted to a hospital in Winnipeg, Manitoba on November 1. He had a history of hypertension for which he was taking ramipril and right arm thrombosis for which he was taking apixaban. 

The patient complained of radicular pain in his arms and legs requiring opioids. He recalled no recent travel, immunizations, or mosquito bites. Physical examination revealed tachycardia, confusion, dysarthria, and difficulty following commands. He did not have fever, rash, or nuchal rigidity. A neurologic examination demonstrated a bilateral intention tremor, twitching, dysmetria, and ataxia. 

Laboratory testing of the patient’s blood samples showed mild hypokalemia and leukopenia (4.1 cells/μL). Magnetic resonance imaging of the patient’s brain revealed a punctate T2 hyperintensity in the right frontal lobe white matter. Electroencephalography revealed mild bilateral fronto-temporal cerebral dysfunction. Cerebrospinal fluid (CSF) examination showed 41 nucleated cells/mm^3^ (89% lymphocytes) and a protein level of 1.41 g/L (reference range 0.2–0.4 g/L); glucose level was within reference range. Results of laboratory testing of the CSF was negative for West Nile virus IgM, Epstein-Barr virus, cytomegalovirus, herpes simplex virus 1 and 2, and varicella zoster virus; bacterial and viral cultures yielded negative results. PCR testing of the CSF was negative for human herpesvirus 6. Additional serum testing was negative for HIV, syphilis, hepatitis B and C, and Lyme disease. PCR testing on a stool sample was negative for enteroviruses. 

We ordered Powassan virus (POWV) testing of convalescent serum, and results were positive for IgM. A 90% plaque reduction neutralization test (PRNT_90_) resulted in antibody neutralization at a dilution of 1:80 on November 3 and then 1:160 on November 6. On the basis of clinical symptoms, timeline from tick attachment to symptom onset, and confirmatory PRNT_90_, we made a diagnosis of Powassan encephalitis. After 1 week, the patient improved and was discharged. Repeat serologic testing on July 14, 2023, showed that PRNT_90_ had decreased to 1:20.

POWV is a flavivirus transmitted by tick species that also act as reservoirs (*1*). The most consequential vectors are black-legged ticks, which are known to bite humans and can spread other tickborne pathogens such as *Borrelia burgdorferi* (Lyme disease), *Anaplasma phagocytophilum* (anaplasmosis), and *Babesia microti* (babesiosis) (*2*). Those pathogens require tick attachment periods >24 hours (*2*), but according to animal studies, the transmission time of POWV from vector to host can occur in 15 minutes (*2*), although transmission typically occurs after 3 hours in humans (*3*). No human-to-human transmission has been reported.

POWV is found in Canada, the United States, and Russia (*1*). In the northeastern United States, >200 cases have been reported. The highest incidence is in Wisconsin and Minnesota, both bordering Manitoba (*1*,*4*). Cases occur predominantly in May–November, when ticks are active (*4*). Only 21 cases have been reported in Ontario, New Brunswick, and Quebec (*1*), Canada. The true prevalence in Canada is unknown because POWV is not a reportable disease. Serologic surveys from 1968–1969 in British Columbia found antibodies in 0.129% of those tested and higher rates of 12.4% in outdoor workers (*5*). Studies in Ontario from the 1970s found antibodies in 0.70% of persons tested (*1*). The range of black-legged ticks is expanding up to 46 km annually, so exposure is likely increasing (*6*). No data on the prevalence of POWV in black-legged ticks in Manitoba have been published.

The incubation period of POWV is 7–34 days, after which 1–3 days of influenza-like prodrome occurs (*7*). Central nervous system infection with encephalitis is common (*7*). During 2011–2020, the United States reported 194 cases; 91.75% were neuroinvasive, and 10%–15% resulted in death (*4*,*7*). Fevers, weakness, headaches, and altered sensorium are the most common patient complaints reported (*7*,*8*). Other complaints include gastrointestinal involvement, focal neurologic signs, seizures, ataxia, twitching, tremors, and radiculitis (*7*). Magnetic resonance imaging findings commonly include T2/flair hyperintensities in the brainstem, cortex, and deep gray structures (*9*). Electroencephalography slowing has been described (*8*). Those findings are corroborated by autopsy results showing high POWV RNA levels in brain tissue (*10*). Neurologic sequelae occur in >50% of survivors. In the case we report, the patient reported persistent ataxia for months. Because no specific antiviral drug is available, disease management consists of supportive measures for airway protection and cerebral edema and analgesia for radiculitis.

A lack of reporting, limited awareness of POWV as a causative agent of encephalitis, expanding tick range, and incomplete knowledge of prevalence has led to a lack of action against this emerging virus. Prevention strategies include avoiding ticks, using insect repellant, treating clothing with 0.5% permethrin in endemic areas, and frequent tick checks.
